# Celastrol Inhibits Lung Infiltration in Differential Syndrome Animal Models by Reducing TNF-α and ICAM-1 Levels while Preserving Differentiation in ATRA-Induced Acute Promyelocytic Leukemia Cells

**DOI:** 10.1371/journal.pone.0105131

**Published:** 2014-08-12

**Authors:** Li-min Xu, Yue-juan Zheng, Ying Wang, Yang Yang, Fan-fan Cao, Bin Peng, Xiong-fei Xu, Hua-zhang An, Ao-xiang Zheng, Deng-hai Zhang, Georges Uzan, Yi-zhi Yu

**Affiliations:** 1 National Key Laboratory of Medical Immunology & Institute of Immunology, Second Military Medical University, Shanghai, China; 2 Sino-French Cooperative Central Lab, Shanghai Gongli Hospital, Shanghai, China; 3 Department of Immunology and Microbiology, Shanghai University of Traditional Chinese Medicine, Shanghai, China; 4 U972, Inserm, Hôpital Paul Brousse, Paris, France; Penn State University, United States of America

## Abstract

All-trans retinoic acid (ATRA) is a revolutionary agent for acute promyelocytic leukemia (APL) treatment via differentiation induction. However, ATRA treatment also increases cytokine, chemokine, and adhesive molecule (mainly ICAM-1) expression, which can cause clinical complications, including a severe situation known as differentiation syndrome (DS) which can cause death. Therefore, it is of clinical significance to find a strategy to specifically blunt inflammatory effects while preserving differentiation. Here we report that the natural compound, celastrol, could effectively block lung infiltrations in DS animal models created by loading ATRA-induced APL cell line NB4. In ATRA-treated NB4 cells, celastrol could potently inhibit ICAM-1 elevation and partially reduce TNF-α and IL-1β secretion, though treatment showed no effects on IL-8 and MCP-1 levels. Celastrol’s effect on ICAM-1 in ATRA-treated NB4 was related to reducing MEK1/ERK1 activation. Strikingly and encouragingly, celastrol showed no obvious effects on ATRA-induced NB4 differentiation, as determined by morphology, enzymes, and surface markers. Our results show that celastrol is a promising and unique agent for managing the side effects of ATRA application on APL, and suggest that hyper-inflammatory ability is accompanied by, but not necessary for, APL differentiation. Thus we offered an encouraging novel strategy to further improve differentiation therapy.

## Introduction

Differentiation inductions by all-trans retinoid acid (ATRA) have revolutionized outcomes of acute promyelocytic leukemia (APL) [1]–[3]. Yet, during the differentiation process, APL cells, in an undefined way, increase cytokine and chemokine secretion and adhesive molecule (mainly ICAM-1) expression, resulting in excessive inflammation. This may cause clinical problems including a severe situation known as differentiation syndrome (DS) that has not been fully addressed [4]. Therefore, to find a novel way to blunt this differentiation-accompanied hyper-inflammation while still preserving induction ability is of clinical significance.

In clinical differentiation induction application, 80–90% of APL patients suffer from one or more inflammation-related symptoms, such as respiratory distress/pulmonary infiltration, fever pleural effusion, renal failure, pericardial effusion, cardiac failure, hypotension, *etc*. About one quarter of patients simultaneously show three or more of these symptoms, a situation previously diagnosed as retinoic syndrome, and now called DS [5]. Through more than twenty years’ practice experience, mainly through close observation and prompt application of corticosteroids, mortality associated with DS has been reduced from its historical high of 30%, but approximately 2–10% of patients still die from DS today [5], [6].

Considering that the physiological maturation of polynuclear white blood cells shows only background level of inflammatory cytokine synthesis and adhesive molecule expressions, we thought that hyper-inflammatory ability might accompany, but not be necessary for, ATRA-induced APL cell differentiation. In this sense, we look for agent(s) that can specifically blunt APL’s problem-causing hyper-inflammatory arm while preserving its beneficial differentiation arm in induction.

Two facts lead us to think that celastrol, a triterpenoid compound first extracted from the Chinese herb *tripterygium wilfordii* Hook f, might be an agent worth exploration. First, we and others have proved that celastrol can strongly inhibit inflammation and its related molecules, such as TNFα, IL-1β, ICAM-1, etc., in various situations [7]–[9], and second, there are reports that triterpenoid compounds can induce differentiation in some leukemia cells [10], [11]. Such expectancy is encouraged by our pre-experiment, which showed that celastrol could inhibit ATRA-caused ICAM-1 expression [12]. This is of importance considering that ICAM-1 plays a fundamental role in ATRA treatment-caused hyper-inflammation [13], [14].

In this study, we test whether or not celastrol is a viable agent for use as mentioned above. We first observed the effects of celastrol on lung infiltrations in DS animal models made by loading ATRA-treated NB4 cells, and then detected the effects of celastrol on pro-inflammatory molecules related to DS, *i.e.*, cytokines TNFα and IL-1β, chemokines IL-8 and MCP-1, and the adhesive molecule ICAM-1 in ATRA-induced NB4. The way in which celastrol affects ICAM-1 elevation was also investigated. Finally, we detected celastrol’s effects on ATRA-induced differentiation.

## Materials and Methods

### Reagents and drugs

RPMI 1640, fetal bovine serum (FBS), penicillin, and streptomycin were obtained from GE healthcare (PAA, Pasching, Austria); Anti-CD54-FITC was purchased from Biolegend (San Diego, CA, USA). Anti-CD13-FITC, CD33-PE, anti-CD15-PE, and anti-CD11b- PE were purchased from Affymetrix (eBioscience, San Diego, CA, USA). Primary antibodies for p-MEK1/2, total MEK1/2, p-P44/42 MAPK, total P44/42 MAPK, p-JNK, total JNK, p-P38, total P38, ICAM-1, p-P65, and total P65 were all obtained from Cell Signaling Technology (Danvers, MA, USA). The primary antibodies for p-STAT1 and total STAT1 were purchased from Beckton Dickinson (BD Biosciences, San Jose, CA, USA). Anti-β-actin and horseradish peroxidase coupled secondary antibodies were purchased from Santa Cruz Biotechnology (CA, USA). Horseradish peroxidase coupled secondary antibody was purchased from Cell Signaling Technology (Danvers, MA, USA).

Dimethyl sulfoxide (DMSO), all-trans retinoic acid (ATRA), and celastrol were all purchased from Sigma-Aldrich (St Louis, MO, USA). ATRA and celastrol were dissolved to 100 mM in DMSO, stored at −20°C, and used within three month. ATRA and celastrol was further diluted with culture medium (for cell culture experiments) or PBS (for animal experiments) just before use.

### Cell culture and treatment

APL cell line NB4 was generously provided by Prof. Tong Jian-hua (Shanghai Ruijin Hematology Institute, China), which originated from Dr. M. Lanotte, INSERM Unit 301, St. Louis Hospital (Paris) [15]. This cell line was established from a patient with APL, who harbors characteristic chromosome abnormality, *i.e.*, t(15∶17), and responds to ATRA with granulocytic differentiation. NB4 cells were seeded at a density of 5×10^5^ cells/mL and maintained in RPMI-1640 culture medium supplemented with 10% fetal calf serum, 2 mM of L-glutamine, 100 U/ml penicillin, and 100 µg/ml streptomycin stored at 37°C in a 5% CO_2_ humidified atmosphere.

For experimentation, NB4 cells were plated at 2×10^5^ cells/ml in complete RPMI- 1640 medium and pre-treated with celastrol or protein inhibitors for 30 min, and then incubated in the presence of ATRA (1 µmol/L) for the indicated times. ATRA at 1 µM was used to induce NB4 maturation to granulocytes. P38 inhibitor SB203580, JNK inhibitor SP5600125, and MEK inhibitor U0126 were purchased from Cell Signaling Technology (Danvers, MA, USA). P65 inhibitor BAY11-7802, ERK inhibitor FR180204, and STAT1 inhibitor AG490 were purchased from Merck Millipore (Calbiochem, Darmstadt, Germany).

### Cell enumeration and cell cycle analysis

Cell number was counted by flow cytometry, based on a single-tube platform method with self-made Cell-beads as internal controls, a method originally reported by Harrison *et al* [16] and modified by us [17], [18]. Briefly, after different treatments, cells were collected, well dispersed, and washed with PBS, and then a known number of green fluorescence-containing Cell-beads were added and mixed well. Before the samples were analyzed by flow cytometer, 7-amino-actinomycin D (7-AAD from BD Biosciences) with a final concentration of 1 µg/ml was loaded. Then, the ratio of living and dead (7-AAD positive) cells to Cell-beads was cytometrically detected (FACS caliber, Becton-Dickinson, CA, USA). The absolute numbers of living and dead cells were calculated by multiplying these ratios by the number of Cell-beads added.

### SD mice models and treatment

Seven- to eight-week non-obese diabetic/severe combined immunodeficient (NOD/SCID) male mice (weighting about 20 g) were purchased from National Rodent Laboratory Animal Resources, Shanghai Branch (Shanghai, China), and maintained at Shanghai Shilaike Laboratory Animal Co, Ltd., Shanghai, China. The maintenance of animal and experimental procedures were approved by Shanghai Animal Welfare and Research Ethics Committee, Science and Technology Committee of Shanghai Municipal Government, China, license numbers SCXK(Hu)2007-0005 and SCXK(Hu)2012-0002 (animal experiments were performed for twice, each time 24 mice were used). The animals were kept under specific pathogen-free conditions, with a 12 hour light-dark cycle. Water and food was provided ad libitum. For each experiment, twenty-four mice were randomly divided into four groups to receive different treatments (depicted in the figure of ‘Effects of celastrol on SD mice model lung infiltrations’ in the Result section); (A) non-SD model receiving DMSO, (B) non-SD model receiving celastrol, (C) SD model receiving DMSO, and (D) SD model receiving celastrol treatment. SD mice models were created according to the report of Ninomiya *et al* [19]. Briefly, on the first day, mice were intravenously injected via a tail vein with 1×10^7^ NB4 cells induced by ATRA at 1 µM for 6 d. From days 1 to 6, the mice were orally administered 100 µL of ATRA (1 mg/ml) every day. Non-model mice were intravenously injected via a tail vein with 1×10^7^ NB4 not induced by ATRA, and from days 1 to 6 the mice were orally administered with 100 µL of PBS every day. Celastrol (or control DMSO) administration, either in non-model or model mice was via one intraperitoneal injection of 100 µL of PBS containing 300 µg/ml celastrol (or the DMSO equivalent), from days 1 to 6. On day 7, all mice were sacrificed after anesthesia, body weights measured, organs extracted, and peripheral blood and bone marrow examined. The extracted lungs were fixed with 10% formalin, and 4-µm sections were mounted on slides and stained with hematoxylin and eosin.

### ELISA determination of cytokine levels

After NB4 cells were subjected to different treatments, the culture mediums were recovered by centrifugation at 1,100 g for 10 minutes. The concentrations of four cytokines in the collected medium were determined by the enzyme-linked immunoadsorption assay (ELISA) using commercially available kits according to manufacturer (Dakewei Biotechnology Co., Shenzhen, Guangdong Province, China) instructions. The lowest sensitivity limit for the cytokine assays follows: 1.6 pg/ml for IL-1β, 4.4 pg/ml for TNF-α, 1.6 pg/ml for IL-8, and 1.6 pg/ml for MCP-1.

### Flow cytometric analysis of adhesion molecules and surface differentiation antigens

After being cultured with different conditions, NB4 cells were harvested and re-suspended in 50 µL PBS-0.5% BSA solution containing appropriate concentrations of the antibodies for adhesion molecules or surface differentiation markers. Corresponding isotype antibodies were used as controls. After 30 min incubation at 4°C in the dark, cells were washed twice and analyzed immediately by FACSCalibur cytometer, with data being collected by CellQuest software.

### Western blot detection of signaling molecules

Cells were lysed with lysing buffer, supplemented with protease inhibitor cocktail, and then protein concentrations were measured by BCA assay. Equal amounts of total protein from different samples were subjected to 10% SDS-polyacrylamide gels and then transferred to polyvinylidenedifluoride membranes, which were probed with the indicated primary antibodies. Detection was accomplished using corresponding horseradish peroxidase (HRP)-conjugated secondary antibodies followed by development with ECL Plus (Beyotime Co., Suzhou, Jiangsu Province, China). Images were captured by G: BOX iChemi XR (Syngene Inc., UK). The intensities of the bands were analyzed by Image J2x software. Cell lysing solution, protease inhibitor cocktail, BCA protein assay kit, and chemiluminescent substrate were purchased from Thermo Scientific (Pierce, Rockford, MA, USA).

### Quantitative RT-PCR of ICAM-1 mRNA

Total RNA was isolated using TRIzol reagent according to the instructions of the manufacturer (Life Technologies Corporation (Invitrogen, Carlsbad, CA, USA). RNA quantity and quality were confirmed with a Drop-1000 Spectrophotometer (NanoDrop Technologies, Wilmington, USA). cDNA was synthesized from 1 µg total RNA by oligo(dT)18 primers and M-MLV reverse transcriptase. Quantitative RT-PCR analysis was performed using the LightCycler (Roche China Center, Shanghai, China) and Taq DNA polymerase. Specific primers of ICAM-1 used for quantitative RT-PCR analysis assays were 5′ CAC AAG CCA CGC CTC CCT GAACCT A 3′ (sense) and 5′ TGT GGG CCT TTG TGT TTT GAT GCT A 3′ (antisense) [8]. Data were normalized by β-actin level. Oligo(dT)18 primers, Taq DNA polymerase, and M-MLV reverse transcriptase were from Takra Biotechnology Co., Ltd. (Dalian, LiaoNing Province, China).

### Morphologic observation of differentiation

Cellular morphology was assessed by light microscopy of Wright’s-stained cyto-smear preparations as follows: after treatment, cells were pelleted at 1000 rpm for 5 minutes and the supernatant discarded. Cyto-smear for microscopic examination was prepared by spreading a small drop of cell pellet on a glass microscope slide and air-drying. The smears were subjected to Wright’s staining and observed by microscope under high power field. Wright’s staining solution and hematoxylin-eosin staining solution were from Sigma-Aldrich.

### NBT reduction analysis

After treatment, NB4 cells were washed and then reconstructed with 100 µL serum-free RPMI-1640 medium containing 2 mg/ml of nitroblue tetrozolium (NBT, Sigma-Aldrich) and 1 mg/ml of 12-ο-tetradecanoylphorbol-13-acetate (PMA, Sigma-Aldrich). The reaction mixture was incubated at 37°C for 1 h. Cell numbers were adjusted to 1×10^6^ for each test before incubation with NBT. The mixture was then treated with 0.04 M HCL 10% SDS for 24 h and the OD measured at a wavelength of 560 nm.

### Statistics

Data in this study are presented as mean ± SE. One-way ANOVA with Dunnet’s or Tukey’s post-hoc test was used for statistical evaluation of significant differences among the groups. A value of *P*<0.05 was considered to be statistical significance. Experiments were repeated at least three times.

## Results

### Celastrol blocked DS model lung infiltrations created by loading ATRA-treated NB4 cells

Ninomiya *et al* created DS in NOD/SCID mice by injecting ATRA-treated NB4 and continuous loading of ATRA [19]. To test the efficacy of celastrol management of DS, we observed the effects of celastrol on lung infiltrations, a typical pathological feature commonly seen in DS.

**Figure 1:**
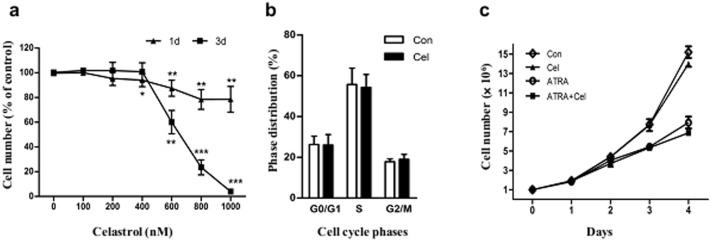
Effects of celastrol on NB4 cells proliferations. (**a**) Dose-effects of celastrol on NB4 numbers. NB4 cells were treated with different doses of celastrol for 1 or 3 d, then the cellular number determined by one-tube platform flow cytometry (detailed in Material and Methods). (**b**) The effects of celastrol on cell cycle distribution in NB4. The cells were treated with celastrol at 200 nM or DMSO for 3d, and then the cell cycles were analyzed with flow cytometry. (**c**) Time-effects of celastrol on cellular number in NB4 co-treated with ATRA. NB4 cells were pre-treated with celastrol at 200 nM (or not) for 30 min, before 1 µM of ATRA was loaded (or not). The cellular numbers at the different indicated time points were determined by one-tube platform flow cytometry. In panels A and B, the data are shown as mean ± SE. * represents *P*<0.05, while ** and *** represent *P*<0.01 and *P*<0.001, respectively, with a sample size (n) of 3.

Before carrying out animal experiment, we at first observed the dose-effects of celastrol on proliferation in NB4 cells induced or not induced with ATRA, thus to select the dose of celastrol used in this study. The results showed that incubation with up to 1 µM celastrol (the maximum dose tested in our study) for 1 d had no obvious effects on cell number. However, when incubation was extended to 3 d, celastrol showed proliferation inhibition at doses equal to or above 400 nM, while 200 nM had no effects on cell numbers (Figure 1a); accordingly, three-day incubation of celastrol at 200 nM showed no obvious effects on cell cycle distribution (Figure 1b). The *in vitro* protocol for inducing NB4 differentiation was carried out by incubating cells with ATRA at 1 µM for 4 days. Our results showed that ATRA induction reduces cellular numbers. We also observed the effects of celastrol at 200 nM on proliferations of cells receiving induction treatment; co-use of celastrol at 200 nM did not show obvious reduction in cell numbers when compared with ATRA alone (see Figure 1c). Since celastrol at 200 nM showed no effect on proliferations in NB4 receiving or not receiving ATRA induction, this dosage was chosen for the following *in vitro* experiments, unless otherwise specified. The molar ratio of celastrol to ATRA was 1∶5, which was used *in vitro* as well as in the following animal experiment.

**Figure 2:**
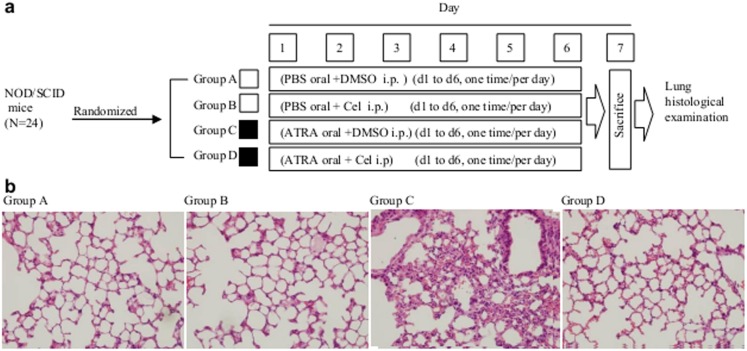
Effects of celastrol on SD mice model lung infiltrations. (**a**) Scheme of experimental design. The 24 NOD/SCID mice were randomly divided into 4 groups receiving different treatments. *i.p.* = intraperitoneal injection. (**b**) Histology examination of lung tissues from NOD/SCID mice with different treatments. After 6 days treatment, the mice were sacrificed and the lungs removed to make tissue sections. These were stained with haematoxylin and eosin and examined by microscope under high power field.

The animal study’s design is depicted in Figure 2a. The NOD/SCID mice receiving a tail vein injection of untreated NB4 with subsequent six-day oral administration of saline and abdominal injections of DMSO or celastrol showed no cellular infiltration in the lungs (Figure 2b, group A and group B), yet, the mice receiving ATRA-treated NB4 and subsequent six-day oral administration of 100 µl of ATRA at 1 mg/ml (DS model) and abdominal injection of DMSO (celastrol’s control) showed severe cellular infiltration in the lungs, this result consistent with another report [19] (Figure 2b, group C). The DS models receiving abdominal injection of 100 µl of celastrol at 300 µg/ml (with the molar ratio of celastrol to ATRA being 1∶5) showed no lung infiltration (Figure 2b, group D), indicating that celastrol could effectively block this pathologic process caused by ATRA-treated NB4.

Animal experiments were performed twice, with 12 animals total in each group, 3 mice died in Group C, 1 in Group D died, and none died in Groups A and B; yet the differences were not statistically significant, perhaps due to small sample size or short observation duration. Celastrol treatment did not cause obvious changes in animal body weight or to internal organs or detract from inhibition when compared with the DMSO control (data not shown).

### Celastrol inhibited ATRA-caused pro-inflammatory cytokine exertion and adhesive molecule expression in NB4 Cells

The *in vivo* results mentioned above encouraged us to explore the possible molecular basis for celastrol’s action, so we observed its effects on pro-inflammatory molecules reportedly involved in DS, including cytokines TNFα and IL-1β, chemokines IL-8 and MCP-1, and the adhesive molecule ICAM-1 in NB4 induced by ATRA.

ATRA at 1 µM could stimulate secretion of TNF-α, IL-1β, MCP-1, and IL-8. Pre-loading 200 nM of celastrol 30 min before ATRA addition significantly decreased TNF-α and IL-1β levels (Table 1); celastrol’s mean inhibition rate for TNF-α and IL-1β in ATRA-treated NB4 after 96 h was 43.7% and 28.1%, respectively. Inhibition of IL-1β was also observed in samples treated with celastrol for 48 h. However, there was no alteration in MCP-1 or IL-8 level with pre-ATRA treatment loading of celastrol (Table 1). Celastrol could reduce pro-inflammatory cytokines TNF-α and IL-1β, but not the chemokines MCP-1 and IL-8.

**Table 1:** The effects of celastrol on secretions of cytokines and chemokines in ATRA-induced NB4 cells.

		Groups (n = 3)			
		Control	Celastrol	ATRA	ATRA+Celastrol
IL-1β	24 h	2.7±0.9	2.4±0.8	2.4±0.7	2.6±0.6
(pg/mL)	48 h	6.4±1.0	4.3±1.2	8.3±0.9	4.4±0.8*
	96 h	6.4±1.0	6.4±2.0	31.6±2.6	22.5±3*
TNF-α	24 h	12.8±0.8	13.1±0.5	7.9±0.4	8.9±0.8
(pg/mL)	48 h	19.7±1.0	17.1±1.1	17.5±1.2	13.1±1.3
	96 h	40.7±2.3	36.1±2.1	648.9±34.0	365.1±23.0**
IL-8	24 h	978.4±18.1	1135.4±160.6	1135.4±320.7	1135.4±242.8
(pg/mL)	48 h	2594.5±89.9	3578.5±256.2	22306.5±783.1	19186.6±543.2
	96 h	1441.6±22.6	1800.5±390.4	27395.1±916.9	27156.1±256.3
MCP-1	24 h	ND	ND	ND	ND
(pg/mL)	48 h	ND	ND	ND	ND
	96 h	908.0±31.2	784.6±22.3	56339.6±1687.3	56206.2±1965.4

Note: * and ** represent *P*<0.05 and *P*<0.01, respectively, when compared with DMSO control; ND means ‘not detected’.

**Figure 3:**
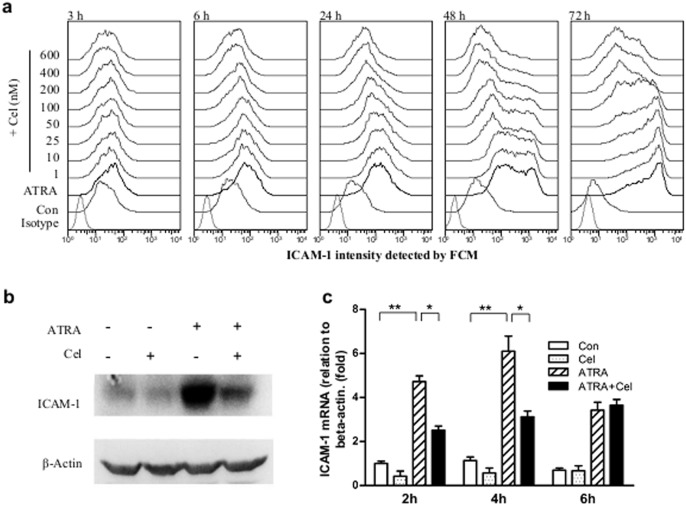
Effects of celastrol on ICAM-1 expressions in ATRA-induced NB4. (**a**) Histograms of ICAM-1 expressions in NB4 cells with different treatments. NB4 cells were treated with celastrol at different doses for 30 min prior to loading 1 µM of ATRA. Cell surface levels of ICAM-1 were detected by flow cytometry at the indicated times after co-culture, with the ATRA loading time as the starting point. (**b**) Western blot detection of total amount of ICAM-1 in cells. NB4 cells were pre-treated with celastrol at 200 nM for 30 min, then 1 µM of ATRA loaded. The culture process continued for 6 hours and then the whole protein was extracted and ICAM-1 detected by Western blot. β-actin was used as internal control. (**c**) Quantitative RT-PCR detection of ICAM-1 mRNA in NB4. NB4 cells were pre-treated with celastrol at 200 nM for 30 min, then 1 µM of ATRA loaded. The culture process continued for the indicated times, after which total RNA was extracted and ICAM-1 mRNA detected with quantitative RT-PCR. β-actin was used as internal control. The data are shown as mean ± SE, * represents *P*<0.05, while ** represents *P*<0.01. Sample size (n) was 3.

In our pre-experiment, we found that celastrol (at 200 nM) could inhibit surface ICAM-1 expressions in NB4 treated with ATRA for 24 h [12]. In these tests we expanded the range of ATRA incubation times from 3 h to 72 h. Flow cytometric detection showed that ATRA-caused surface ICAM-1 elevation could be observed as soon as 3 h after incubation, and continued to rise upward towards 72 h. When celastrol was pre-loaded for 30 min, the inhibitory effect toward ATRA-induced ICAM-1 elevation was observed at all of the time points; these inhibitory rates increasing with incubation time and dose (Figure 3a). For example, the mean inhibition rate of celastrol at 200 nM for ATRA-induced NB4 at 3 h, 6 h, 24 h, 48 h and 72 h were 41, 51, 66, 72 and 80%, respectively, while these values for celastrol at 600 nM (also at 3, 6, 24, 48, and 72 h) were 49, 67, 79, 85 and 88%, respectively.

Since ICAM-1 has been reportedly to be important for developing DS [13], [14], we further investigated at which level celastrol to exert its inhibitory effects on ICAM-1 expressions. In addition to inhibiting ICAM-1 surface levels, Western blot results showed that celastrol pre-loading for 30 min could dramatically inhibit whole cell (surface and intracellular) ICAM-1 elevation caused by 24 h ATRA induction (Figure 3b). Furthermore, quantitative RT-PCR results showed elevated ICAM-1 mRNA at 2 h, 4 h, or 6 h after ATRA treatment, with the most significant changes at 2 h and 4 h; pre-loading of celastrol for 30 min could significantly inhibit ATRA-caused ICAM-1 mRNA elevation at the 2 h and 4 h time points (Figure 3c). Therefore, celastrol inhibited ICAM-1 transcription, though whether it also affects translation or post-translational translocation needs further investigation.

### Celastrol’s inhibition of ATRA-caused ICAM-1 elevation was related to MEK1/ERK1 activation

The above results indicated that celastrol could blunt excessive ATRA induction-caused inflammation and inhibit related molecules, especially ICAM-1. An interesting issue, which pathway might be related to celastrol’s inhibition of ICAM-1, thus attracted our attention. To address it, we observed alterations of the main signaling molecules already reported as celastrol’s targets, including MEK1, ERK1, NF-κB, JNK, p38, and STAT1 [8], [20]–[24] in cells treated with ATRA and celastrol, and these agents’ roles in celastrol’s ICAM-1 inhibition.

**Figure 4:**
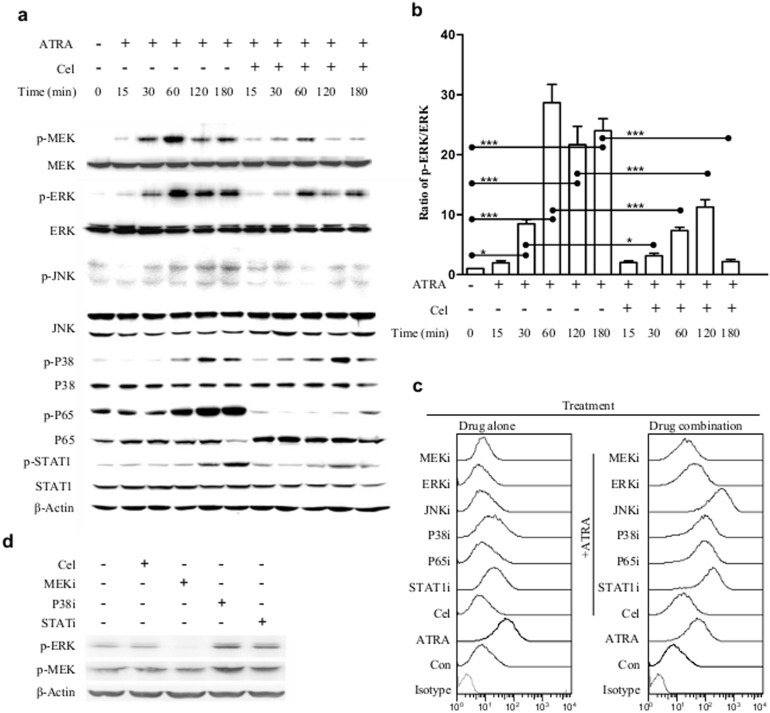
Relationship of a group of signaling proteins and the effects of ATRA/celastrol on ICAM-1 expressions in NB4. (**a**) The protein levels and activations for a group of signaling proteins in NB4 cells treated with ATRA or celastrol plus ATRA for different time periods. NB4 cells were pre-treated or not with celastrol for 30 min, then 1 µM ATRA added for different time periods (with ATRA addition as the starting point). The total protein was extracted and the level and phosphorylation (activation) of a group of signaling proteins were detected with respective antibodies via Western blot. (**b**) Time-dependent alterations of the ratios of p-ERK/ERK. The bands of the Western blot as shown in figure (a) were scanned and the ratios of band intensity were calculated and compared. (**c**) The effects of several signaling inhibitors and celastrol on ICAM-1 expressions in NB4. NB4 cells were pre-treated or not with celastrol or with one of the signaling inhibitors for 30 min, then 1 µM ATRA added for 24 h. The cells were then harvested and surface levels of ICAM-1 detected by flow cytometry. (**d**) The effects of signaling inhibitors on MEK1/ERK1 activation. NB4 cells were treated or not with celastrol or one of the signaling inhibitors for 30 min, and the levels and activations of MEK1 and ERK1 were detected with Western blot. The data are shown as mean ± SE, * represents *P*<0.05, while *** represents *P*<0.001, the sample size (n) was 3.

ATRA treatment can activate MEK1 and its downstream target ERK1, as well as p38, NF-κB(p65), and STAT1, while only slightly activating JNK, as determined by the phosphorylation of these signaling proteins. Pre-loading celastrol for 30 min could inhibit the actions of MEK1, ERK1, NF-κB (p65), and STAT1, but not p38 (Figure 4a and 4b). These results suggest that MEK1, ERK1, NF-κB (p65), and STAT1 are prime candidates for those involved in celastrol’s ICAM-1 inhibition. However, of these, only the MEK1 and ERK1 inhibitors could significantly inhibit ATRA-caused ICAM-1 elevation (Figure 4c), with the MEK1 inhibitor showing stronger inhibition (mean inhibition rates were 59% for MEK1 inhibitor and 31% for ERK1 inhibitor). Therefore, MEK1 and ERK1, particularly MEK1, is important to ATRA-induced ICAM-1 expression and to celastrol’s action.

To further support the importance of MEK1/ERK1 in our system, p38 or STAT1 inhibitors could further raise ICAM-1 expressions in non-induced or ATRA-induced NB4 (Figure 4c). These two inhibitors also increased MEK1 and ERK1 activation in NB4 (Figure 4d).

### Celastrol did not affect ATRA-induced differentiation of NB4 cells

The ultimate goal of our research was to identify an agent that could blunt excessive inflammation while preserving differentiation in ATRA-treated APL; hence, after confirming the inhibitory effects of celastrol on inflammation and its relevant pathways, we began to observe celastrol’s effects on differentiation.

**Figure 5:**
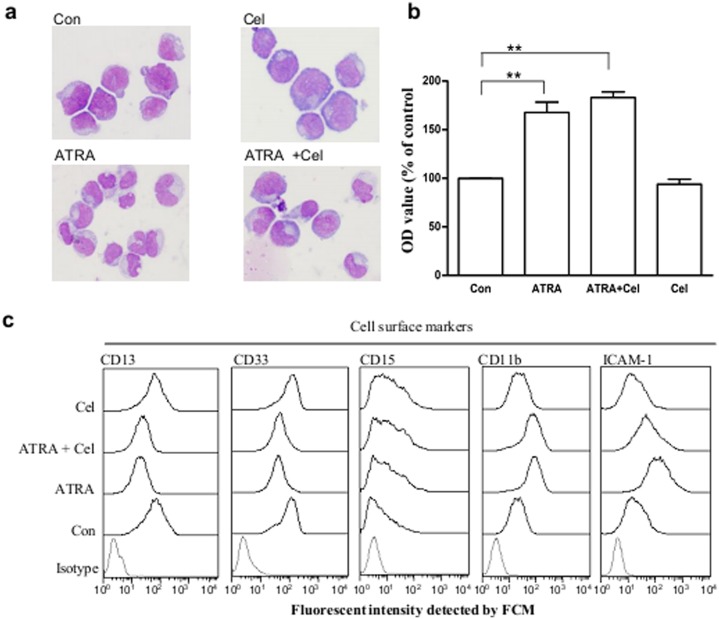
Effects of celastrol on ATRA-induced differentiation in NB4. (**a**) Morphologic observations of NB4 cells with different treatments. NB4 cells were pre-treated with celastrol at 200 nM for 30 min, then 1 µM ATRA added for 96 h culturing. Cells were subjected to Wright’s staining before examination with microscope under higher power field. (**b**) NB reduction ability in different cells. Cell treatment is the same as in panel A. NB reduction ability was determined by colorimetric method. Data are reported as mean ± SE, with a sample size of three. (**c**) Cell surface markers in NB4 cells with different treatments. Cell treatment is the same as in panel A. After treatment, the surface markers in NB4 were detected by flow cytometry. The data are shown as mean ± SE, ** represents *P*<0.01, with a sample size (n) of 3.

Morphologic observations are still the main means for differentiation evaluation. Morphological alterations in NB4 cells were followed after incubation with ATRA, celastrol, or a combination of the two. After incubation with ATRA for 96 h, NB4 cells aggregated and the nuclei became irregular, showing differential features. Pre-loading celastrol for 30 min had no obvious effects on these ATRA-induced morphological changes (Figure 5a).

Engulf ability increases with granulocyte maturity, and NBT reduction ability, a representative of the engulf ability, is often used in differentiation evaluation. After ATRA treatment for 96 h, NBT reduction ability in NB4 cells increased by 123.8% when compared to the non-treated control. In the celastrol pre-loading group (30 min before ATRA addition), NB4 cells’ increased ability in reducing NBT was not inhibited, but rather slightly, though not significantly, augmented (Figure 5b). Celastrol alone showed no effects on NB4’s NBT reduction ability.

Since the cell maturation process is accompanied by alterations of surface markers, these features may be used to evaluate differentiation. After induction with ATRA at 1 µM for 96 h, NB4 cells decreased their surface expression of CD13 and CD33 and increased expressions of CD15 and CD11b. Celastrol addition 30 min prior to ATRA loading had no obvious effects on ATRA-induced alterations of these four markers, a striking result when compared to celastrol’s potent inhibition towards ATRA-induced ICAM-1 elevation (Figure 5c). Celastrol alone had no obvious effects towards the four markers excerpt a slight elevation of CD11b.

The above results indicate that celastrol has no obvious effects on ATRA-induced NB4 differentiation.

## Discussion

APL cells, when treated by ATRA, increase their pro-inflammatory cytokines and chemokines secretion, as well as adhesive molecules expression, thus causing the clinical complication differentiation syndrome (DS) in some APL patients receiving ATRA treatment. In this work, we found that the natural compound celastrol could effectively block lung cell infiltrations in DS animal models, and accordingly inhibit ATRA-caused elevation of cytokines and adhesive molecules, especially ICAM-1, in NB4. The mechanism for this action was explored and found to be related to MEK1/ERK1 activation reduction. More promising and exciting, celastrol showed no obvious effects on ATRA-induced differentiation.

The most severe clinical situation arising from ATRA treatment is DS resulted from excessive inflammation. By testing a reported animal model of DS syndrome [19], we found that celastrol could reduce cell infiltrations in lungs, a typical pathological feature of DS. Thus we offer *in vivo* support for using celastrol to counteract the excessive inflammation caused by ATRA-induced APL.

Induced APL uses three main kinds of molecular weapons to build up excessive inflammation: cytokines, chemokines, and adhesive molecules. To support our *in vivo* finding, co-incubation of celastrol with ATRA application could significantly reduce ATRA-triggered TNF-α and IL-1β elevation, though no celastrol-caused effects on chemokines MCP-1 and IL-8 were observed.

In addition to affecting cytokines, celastrol had potent inhibition effects on ATRA-caused ICAM-1 expression. Such action could be observed as early as 3 h and increased with time, at least until 72 h (the longest time period we observed). ATRA could elevate ICAM-1 mRNA expression, which celastrol could inhibit. Two other commonly observed adhesive molecules, VCAM-1 and E-selectin were only slightly elevated by ATRA, which were also inhibited by celastrol (data not shown). Some researchers have highlighted the importance of ICAM-1 in building up excessive inflammation in DS. Dore et al reported that DS syndrome was related to some genotype with ICAM-1 [13], and Cunha De Santis et al found that ICAM-1 gene knockout abolished ATRA-caused inflammation-related syndrome in mice [14]. In light of this information and our own observations, we believe that ICAM-1 inhibition may be the major factor in celastrol’s action of preventing cell infiltration in lungs, though reductions in cytokines secretion may also contribute to this result.

The importance of ICAM-1 in building up excessive inflammation (as mentioned above), and celastrol’s potent inhibition effects on its expression, inspired us to search for the signaling molecule responsible for celastrol’s ICAM-1 inhibition. Several signaling molecules reportedly targeted by celastrol [8], [20]–[24] were thus screened. ATRA could activate MEK1, ERK1, NF-κB, JNK, p38, and STAT1, and celastrol could inhibit all these molecule activations except for p38. Yet, since only the MEK1 or ERK1 inhibitor could inhibit ATRA-caused ICAM-1 elevation, we thought that these two proteins were related to ATRA-activated ICAM-1 alterations while being inhibited by celastrol. The finding that the STAT1 or p38 inhibitor could further increase ATRA-caused ICAM-1 expression in NB4 and that these two inhibitors also increased MEK1 and ERK1 activation also supports our notion. There are reports that ATRA can activate MEK1/ERK1 signaling [25]–[27], and that celastrol can inhibit this pathway [28], [29].

MEK1 is upstream from and the main activator of ERK1, yet we found that the MEK1 inhibitor was more effective than the ERK1 inhibitor in suppressing ATRA-caused ICAM-1 elevation, suggesting there may be some way other than MEK1 using ERK1 to regulate ICAM-1 expressions. This is still unclear and needs further investigation. It is also worth noting that there might be at least two ways for celastrol to inhibit ERK1 activation; one is through MEK1 inhibition, another is through direct ERK1 inhibition, a reported action found by informatics simulation and laboratory tests [21].

Celastrol’s action is generally attributed to its NF-κB [30] inhibition; yet in our system, though NF-κB inhibition was observed, it did not contribute to celastrol’s action in lowering ATRA-caused ICAM-1 elevation. HSP90 inhibition is also considered fundamental to celastrol’s action [31], however, celastrol did not cause HSP70 elevation (a hallmark of HSP90 inhibition) in NB4 treated or not treated by ATRA (data not shown). It is unlikely that celastrol’s action was related to HSP90 inhibition in our system.

The most exciting discovery of our work was that celastrol showed no obvious effects on ATRA-induced NB4 differentiation. After incubation with ATRA, NB4 cells displayed maturity characteristics determined by morphological, histological, or biochemical means, and these maturation changes were still observable in groups pre-loaded with celastrol. Therefore, we found celastrol is a novel agent for improving ATRA treatment, in that differentiation-based therapeutic effects are preserved while side effects caused by excessive inflammation are moderated. Our results also suggest that in acquiring hyper-inflammatory capacity, ICAM-1 elevation and TNF-α and IL-1β secretion are more likely to be accompanying rather than necessary events in ATRA-induced APL differentiation. Whether chemokines are needed for differentiation remains an open question and thus needs further investigation. Nevertheless, to be able to separate between the excessive inflammation arm and the maturity signaling arm should encourage researchers to explore other novel agents in addition to celastrol that could specifically blunt unwanted hyper-inflammation in APL differentiation induction.

Here, we provide the first documentation that celastrol can blunt tissue infiltration in DS animal models, reduce pro-inflammatory cytokine secretion, and specifically inhibit the key hyper-inflammation component ICAM-1 in ATRA-induced NB4 in a MEK1/ERK1-related way, all without obvious effects on differentiation. Thus we provide a novel strategy for controlling the side effects of ATRA application in APL therapy.
